# Antenatal Doppler ultrasound implementation in a rural sub-Saharan African setting: exploring the perspectives of women and healthcare providers

**DOI:** 10.1186/s12978-021-01233-5

**Published:** 2021-10-07

**Authors:** Sam Ali, Olive Kabajaasi, Michael G. Kawooya, Josaphat Byamugisha, David Zakus, Aris T. Papageorghiou, Kerstin Klipstein-Grobusch, Marcus J. Rijken

**Affiliations:** 1 Department of Research, Ernest Cook Ultrasound Research and Education Institute (ECUREI), Mengo Hospital, Sir Albert Cook Building, Albert Cook Road, P.O. Box 7161, Kampala, Uganda; 2 School of Medicine, Makerere University College of Health Sciences, Kampala, Uganda; 3 Julius Global Health, Julius Center for Health Sciences and Primary Care, University Medical Center Utrecht, Utrecht University, Utrecht, The Netherlands; 4 Training and Social Sciences Unit, Walimu, Kampala, Uganda; 5 Division of Clinical Public Health, Dalla Lana School of Public Health, University of Toronto, Toronto, Canada; 6 Nuffield Department of Women’s and Reproductive Health, John Radcliffe Hospital, University of Oxford, Oxford, UK; 7 Division of Epidemiology and Biostatistics, School of Public Health, Faculty of Health Sciences, University of the Witwatersrand, Johannesburg, South Africa; 8 Department of Obstetrics and Gynecology, University Medical Center Utrecht, Utrecht University, Utrecht, The Netherlands

**Keywords:** Pregnancy, Antenatal care, Healthcare workers, Doppler ultrasound, Low- and middle-income countries, Qualitative

## Abstract

**Background:**

The World Health Organization recommends research to evaluate the effects of a single third trimester Doppler ultrasound examination on preventable deaths in unselected-risk pregnancies, particularly in low- and middle-income countries (LMICs) where the evidence base is scarce. While evaluating such technologies, researchers often ignore women and health care provider perspectives. This study explored the views and experiences of women and healthcare providers regarding the use of advanced ultrasound technology to optimize the health of mothers and their babies in a rural community in mid-western Uganda.

**Methods:**

We enrolled 53 mothers and 10 healthcare providers, and captured data on their perceptions, barriers, and facilitators to the use of Doppler ultrasound technology using focus group discussions, semi-structured interviews and observations. Using qualitative content analysis, we inductively coded the transcripts in ATLAS.ti 8.0, detecting emerging themes.

**Results:**

Women were afraid that ultrasound would harm them or their fetuses and many of them had never seen an ultrasound scan. The majority of the women found their partners supportive to attend antenatal care and use ultrasound services. Healthcare providers in Kagadi Hospital were unfamiliar with Doppler technology and using it to guide clinical decisions. Other barriers to the implementation of Doppler ultrasound included shortage of trained local staff, insufficient equipment, long distance to and from the hospital, and frequent power cuts.

**Conclusions:**

We found limited exposure to Doppler ultrasound technology among women and healthcare providers in mid-western Uganda. Engaging male partners may potentially influence the likelihood of accepting and using it to improve the health of women and their fetuses while wide spread myths and misconceptions about it may be changed by community engagement. Healthcare workers experienced difficulties in offering follow-up care to mothers detected with complications and Doppler ultrasound required a high level of training. While introducing advanced ultrasound machines to weak health systems, it is important to adequately train healthcare providers to avoid inappropriate interventions based on misinterpretation of the findings, consider where it is likely to be most beneficial, and embed it with realistic clinical practice guidelines.

**Supplementary Information:**

The online version contains supplementary material available at 10.1186/s12978-021-01233-5.

## Plain Language Summary

Globally, nearly three million babies are stillborn every year, but most especially in low- and middle-income countries like Uganda. One of the factors contributing to a high number of stillbirths in low-income countries is the difficulty in identifying complications and accessing high quality care during pregnancy. Although antenatal Doppler scans are being widely used to diagnose complications in high-risk pregnancies in developed countries, studies evaluating it in LMICs are needed before it is implemented on a wide scale. We engaged 53 mothers, eight health workers from a hospital and two healthcare managers from a local government in Uganda to attain their opinions about Doppler ultrasound. We found that spousal involvement may promote acceptance and use of ultrasound services. However, the health workers did not have adequate knowledge about Doppler technology and using it for the benefit of mothers and the mothers feared that ultrasound procedures might harm them or their unborn babies. Making matters worse, the hospital faced frequent power cuts that affected the use of the equipment. Further, mothers must cover a long distance to access the hospital and its services. To reduce the number of babies dying during pregnancy or a few days after birth in Uganda and similar low-resource settings using Doppler technology, it is essential to strengthen the health systems. Starting with the training of healthcare providers to equipping and stabilizing power supply in health facilities, and educating the public about critical health procedures to break myths and misconceptions.

**Supplementary Information:**

The online version contains supplementary material available at 10.1186/s12978-021-01233-5.

## Résumé

**Introduction:**

L’Organisation Mondiale de la Santé encourage la recherche sur l’échographie Doppler unique au troisième trimestre de grossesse et son impact sur la réduction des décès évitables chez les femmes enceintes non sélectionnées, particulièrement dans les pays à bas et moyen revenus où les évidences sont rares. Cette étude a exploré les opinions et expériences des femmes enceintes et des prestataires sur l’utilisation des technologies ultrasonographiques avancées dans une communauté rurale du mid-ouest Ougandais.

**Méthodes:**

Nous avons inclus 53 mères et 10 prestataires de santé, et documenté leurs opinions, les barrières et facilitateurs de l’utilisation de l’échographie Doppler via des entretiens semi-structurés de groupe et individuels et des observations. L’analyse de contenu à partir des transcripts codés de façon inductive dans ATLAS.ti 8.0, ont permis l’identification des thèmes principaux.

**Résultats:**

Les femmes craignaient pour elles et pour leur foetus les lésions que causeraient l’échographie et la plupart n’avaient jamais vu d’échographe. La majorité des femmes avaient le soutien de leurs partenaires pour bénéficier des soins anténatals et passer une échographie. Les prestataires du “Kagadi Hospital” avaient étaient peu sensibilisés de la technologie Doppler et de son utilisation dans la décision clinique. Les autres barrières à l’implémentation de l’écho Doppler incluaient le manque de compétences du staff local, les équipements insuffisants, la distance pour accéder à l’hôpital, et les fréquentes coupures d’électricité.

**Conclusions:**

Les femmes et les prestataires de soins dans le mid-ouest Ougandais sont faiblement exposés à l’échographie. Impliquer les partenaires masculins permettrait d’augmenter son acceptabilité et utilisation et ainsi améliorer la santé des femmes et des bébés. Aussi, des activités d’éducation communautaire contribueraient à modifier les mythes largement propagés et les conceptions erronées sur l’écho Doppler. Par ailleurs, les prestataires font face à des difficultés dans le suivi des femmes présentant des complications, et le Doppler requiert des compétences de haut niveau. L’introduction de technologies avancées d’échographie dans des systèmes de santé fragiles doit tenir compte de l’impact attendu, et nécessite la formation des prestataires afin d’éviter des interprétations biaisées et une prise en charge inappropriée, et leur inclusion dans les guides de pratique clinique.

## Background

In most low- and middle-income countries (LMICs), perinatal mortality rates are very high, about 10 times that of high-income countries (HICs) [1, 2]. In Uganda, the stillbirth rate is 17.8 per 1000 live births [2]. The causes of perinatal mortality in LMICs are complex, ranging from high rates of home deliveries to poor quality of intrapartum care, and undetected pregnancy complications such as growth-restricted fetuses, among others. Significant mortality reductions will require a multi-sectoral effort, including strengthening health facility surveillance systems to accurately detect all fetuses truly at risk of adverse outcomes combined with timely effective medical interventions where the maternal and fetal benefits and risks of such interventions were carefully evaluated. Doppler ultrasound is a potentially valuable surveillance tool and its role in high-risk pregnancies is already established in HICs [3, 4]. In low-risk or unselected obstetric populations, there is still no evidence to indicate that its use improves perinatal outcomes in both LMICs and HICs [4]. In fact, there is a potential risk of inappropriate interventions such as cesarean sections and adverse effects [5]. Its use remains reserved for the fear of high false positive diagnosis and iatrogenic morbidity or even mortality [6]. Studies evaluating the effects of introducing Doppler ultrasound into antenatal care (ANC) on perinatal mortality are needed, especially in low-resource settings where evidence about the effects of this intervention is acutely lacking. While evaluating such technologies, researchers often ignore women and healthcare provider perspectives. Stakeholder perspectives are vital to guide its integration into routine antenatal care to optimize the health of pregnant women and their fetuses.

Previous studies in LMICs have reported experiences and views of local stakeholders on the implementation of obstetric ultrasound [7–13]. In general, pregnant women had mixed views regarding antenatal ultrasound: the majority saw it as beneficial, while some feared it [9–11, 14]. For example, studies in Uganda and Thailand showed that some respondents believed ultrasound could be dangerous [11, 15]. Healthcare providers, meanwhile, perceived it as a useful tool in pregnancy [8, 10, 11]. Furthermore, both healthcare providers and local women reported lack of equipment, personnel capacity, equipment cost and maintenance as major challenges to regular use of ultrasound [12–14]. These findings mostly relate to the use of basic ultrasound equipment. To date, we know little about potential dilemmas in implementing more advanced ultrasound technologies with Doppler and color flow capabilities in LMICs.

This study explored the views and experiences of mothers, healthcare workers, and health system managers regarding the use of Doppler ultrasound in pregnancy in a rural community in mid-western Uganda. We highlight operational and practical issues regarding its clinical application in Uganda and examine the factors that could influence its introduction into similar LMICs.

## Methods

### Design and setting

This explorative qualitative study was conducted and reported per the consolidated criteria for reporting qualitative research (COREQ) [16]. It was part of the EPID project, a larger cohort study involving over 1239 pregnant women prospectively enrolled between 2018 and 2020 in Uganda to evaluate the prognostic accuracy of Doppler ultrasound for adverse perinatal outcomes. The EPID study women underwent pregnancy dating ultrasound scans at < 24 weeks, followed by biometry and Doppler ultrasound examinations in the late third trimester. They were scanned at Kagadi Hospital; a 150-bed health facility located in the greater Kibaale region in mid-western Uganda approximately 215 km from the national capital, Kampala. According to the Uganda Demographic and Health Survey report 2016, the total population for Kibaale stood at 788,714, with 389,278 (49.36%) males and 399,436 (50.64%) females [17]. The district had 168,358 households, giving an average size of 4.7 persons per household. The District Health Information Systems shows that Kagadi hospital had about 4449 deliveries in 2020, and about 1483 women achieved four or more ANC contacts while only 13 achieved eight contacts.

### Sample and recruitment

We recruited women prospectively to a point of saturation of the themes both from the overall EPID study cohort and from the routine antenatal clinic setting in order to ensure that women whom we did not enroll in the EPID study were also represented. Women attending routine antenatal care appointments were recruited in an unselected fashion by convenience sampling: with a quota of one daily. We purposively sampled women recruited from the overall EPID study cohort and healthcare providers.

All participants including emancipated minors and adults 18 years and above provided informed written consent before enrollment. The illiterate participants provided a thumbprint and their interviews were conducted in the local language. The research team assured participants of confidentiality and privacy and promised to act in the spirit of the informed consent received. The study received ethical clearance from Makerere University School of Medicine Research and Ethics Committee (SOMREC): approval number #REC REF 2018-090; and from the Uganda National Council for Science and Technology (UNCST): approval number HS 2459. Further, we obtained written permission from Kagadi district and hospital authorities to implement the study in their territory.

To determine sample size, we looked at previous studies assessing perceptions of key stakeholders on antenatal ultrasound and estimated that approximately 45 participants should be sufficient [10, 11]. However, we recruited the study sample beyond the estimated mark. The study team recruited nearly half of the estimated participants, undertook a preliminary analysis and based on the initial analytic ideas further enrolled more women to a point where no new sub-themes, themes or insights to the research questions emerged.

### Interviews

Data collection methods included focus group discussions (FGDs), semi-structured interviews, observations using a daily activity log, and field notes. We conducted nine FGDs with women (one with women who declined an ultrasound examination, three with pregnant women attending ANC, two with mothers who gave birth at home, and three with those who completed the EPID study follow-up and gave birth in a health facility). Seven individual semi-structured interviews were conducted with women who had been pregnant (three had experienced perinatal death, and four had complications such as ectopic pregnancy, spinal defect, and premature delivery). Ten individual semi-structured interviews were held with eight healthcare workers (two sonographers, five midwives, and one records clerk), and two healthcare managers (administrators of Kagadi hospital and the district health offices) (Table 1).

**Table 1:** Type of interview and participants recruited

Participant categories	Semi-structured interviews	FGD	Number of participants
Women attending ANC		3	18
Women who declined the ultrasound scan examination		1	3
Mothers from EPID study who delivered at home^a^		2	9
Mothers from EPID study who delivered in a health facility^a^		3	16
Mothers who experienced stillbirth or neonatal death^a^	3		3
Mothers who had complications (ectopic pregnancy, spinal defect, preterm deliveries)^a^	4		4
Healthcare workers			
Sonographers	2		2
Nurses and midwives	6		6
District Health Team	2		2
Total	17	9	63

^a^32 participants enrolled from the list of EPID study

We used the interview guides with open-ended questions focused on four main topics: perceptions of women, experiences of women and providers, barriers and facilitators of Doppler ultrasound use in pregnancy to conduct the discussions (Additional files 1, 2, 3 and 4: interview and focus group discussion guides). Each interview began with an explanation of the EPID study, the consent process, and the assignment of unique numbers to FGD participants to assure anonymity. For the women recruited from the EPID study database, we scheduled the interviews at their homes or Kagadi Hospital depending on one’s preference, while all women recruited at the ANC clinic were interviewed at the health facility. We interviewed all healthcare providers at Kagadi Hospital, except for one at the district health offices. The FGDs lasted about 45 minutes and the semi-structured interviews between 20 to 30 minutes. A social scientist (OK) with over 10 years of experience in qualitative research and training in mixed methods research led all the interviews. OK was assisted by two experienced midwives purposively selected from the pool of research assistants serving in the EPID project. They conducted the interviews in English and the local language (Runyoro) between September and December 2019. All interviews were audio-recorded.

### Data analysis

Two study team members (OK and a research assistant) transcribed the audio recordings verbatim and translated them into English. They read the transcripts and listened to the audio recordings simultaneously, to check for accuracy and consistency, resolving disagreements by discussion. The principal investigator (SA) reviewed all the transcripts to ensure reliability before analysis.

Using qualitative content analysis, we inductively coded the transcripts in ATLAS.ti 8. This inductive approach allows for the unexpected and permits more socially-located responses from interviewees that may include matters of cultural beliefs or links to other important events in their lives, such as grief, which cannot be predicted by the researcher in advance [18]. The data analyst (OK) carefully read the transcripts, applying labels (codes) to expressions related to the research question. To identify the codes, categories or themes from the transcripts, we used line-by-line scrutiny (repetitions, similarities and differences, indigenous typologies, and transitions) and processing (cutting and sorting, word list and key words in context, and word co-occurrence) techniques. SA went through the coded transcripts and cut out all the quotes that pertained to each of the major categories.

After the initial analysis, a larger research team (SA, OK, KKG, and MJR) discussed and agreed on the working analytical framework based on the codes and categories emerging from the data. The working thematic framework was then applied by indexing all the subsequent transcripts with the categories and codes, taking care to note any new codes or impressions not in the initial set [18]. We then revised the analytical framework to include new and refined codes, and agreed on the groupings of conceptually related codes. We repeated this process until no new codes, themes or insights emerged from the data. The main themes were generated by reviewing the final matrix, defined and illustrated using direct quotations from the participants. We reported the results in a semi-quantitative format using qualifiers: very few (< 10%), some (10–24%), about half (25–49%), majority (50–75%), most (76–89%) and almost all (> 90%), adopted from a previous study by Das et al. [19].

## Results

We recruited 53 women, 32 from the overall cohort of women participating in the EPID project and 21 from the routine antenatal clinic setting. Forty-six women attended FGDs with an average of six individuals per group while seven consented for IDIs. Their median age (range) was 28 (15–42) years, 17 (32.1%) were primigravida and 36 (67.9%) were married (Table 2). All healthcare providers (two males and eight females) participated in the individual interviews. Their median age (range) was 44 (27–54) years, nine were married and only one was single. Three main themes emerged from the data, including safety, resource availability (service availability, technicalities in performing the Doppler exam and follow-up), and partner involvement (Table 3).

**Table 2:** Background characteristics of women interviewed

Variables	Results	
	Women (N = 53)	Health workers (N = 10)
Age (years)		
Median (range)	28 (15, 42)	44 (27, 54)
Gender		
Male	0 (0)	2 (20.0)
Female	53 (100)	8 (80.0)
Gravidity		
1	17 (32.1)	
2	14 (26.4)	
3	10 (18.9)	
≥ 4	12 (22.6)	
Marital status		
Married	36 (67.9)	9 (9.0)
Separated	3 (5.7)	0 (0)
Single	14 (26.4)	1 (10.0)
Education level		
None	2 (3.8)	0 (0)
Primary	30 (56.6)	0 (0)
Secondary	16 (30.2)	0 (0)
Tertiary	5 (9.4)	10 (100)

^a^Results presented as: n (%)

**Table 3:** Themes or categories of interest obtained from the final analytical framework with constituent codes, descriptions of codes, and illustrative quotes

Codes	Description of the codes	Examples of illustrative quotes
Resource availability		
Ultrasound service availability	Text or phrases related access to the pregnancy dating and Doppler ultrasound services when needed by the women	“When I came back, there was no power, so I returned without going to the scan. Since then, I have been coming and finding no power. I don’t know if I will get a chance of getting scanned today” [**R5: FGD 1 with mothers undergoing routine ANC**] “But the way I see, people really like the scan, they like coming here, even today, mothers came for Doppler and we told them we are sorry that our machine went for service” [**IDI with Sonographer 2**] “Yes, they told me to go back on 10th of July 2019 but when I went there, they told me that the scan is dead and we came back” [**IDI with mother who suffered a neonatal death**]
Doppler exam technicalities	Text describing the Doppler ultrasound examination procedure, including the ease or complexity of performing it and technical limitations of the procedure	“I’m not technical in these things of the scan, but what I have seen when they are doing a Doppler, they take a lot of time…dating is okay but when they are doing Doppler, it takes a lot of time” [**IDI with midwife 5**] “Sometimes also, it takes time to get the vessels, as you are getting there, a mother says am tired, you wait and I change, like that. Though others are fast and they don’t take a lot of time” [**IDI with Sonographer 1**]
Follow-up of pregnant women	Phrases describing different aspects of the follow-up antenatal care, including follow-up Doppler ultrasound services for pregnant women after initiation of the antenatal care visits	“Some mothers have no contact” [**IDI with midwife 5**] “Another thing, may be, is that these mothers complain of distance if you tell them to come back for Doppler” [**IDI with midwife 1**] “Those who don’t have phones and their husbands don’t have, we ask for the chairman’s phone who can deliver reminder information and we tell her that we need you on this date” [**IDI with midwife 3]**
Partner involvement		
Male partner participation	Male partner accompanying his wife or female partner to antenatal care, providing social economic support and ensuring that all recommendations made at the antenatal care are observed to safeguard the wellbeing of the couple and the baby	“Like me, I was with my husband and they told me to go for scan. He also accepted and I came” [**R5: FGD 1 with mothers who delivered in the hospital**] “Me, when I heard about the service, I told him and we started from there to come together [**R0: FGD 3 with mothers who delivered in the hospital**] “I came with my husband and I told him immediately after the scan about the baby. And when we reached home, we explained to my mother-in-law about all the results they gave us” [**R3: FGD 2 with mothers who delivered from home**] “You find you think about going to the hospital, even me I wouldn’t have wished to go to scan late but because you find you are with a man and you don’t agree with each other” [**IDI with mothers who suffered a stillbirth**]
Safety		
Safety of ultrasound	Potential harm to the mother or fetus resulting from exposure to the ultrasound during fetal life, as perceived by the pregnant women	“I fear the scan, if I don’t have a very serious disease, I can never go for a scan. Because they say that the scan reduces peoples’ years” [**R3: FGD 2 with mothers attending routine ANC**] “The truth is, I know very well that when you put your child in the scan, the child won’t be delivered like a clever child. There is a way that electricity affects the child’s brains and he or she becomes dull. So, I said to myself, instead of delivering of a dull child, I would rather not go for the scan” [**R1: FGD with mothers who declined an ultrasound examination**] “Most people here associate the scan with reducing years (life expectancy)” [**Interview: midwife 5**] “Sometimes they tell you, it reduces our age” [**Interview: Sonographer 1**] “Most women in the villages dislike this scan. They think that the scan chops their years” [**Interview with midwife 1**] “I had a lot of thoughts but when they educated me about them, I stopped” **[Interview with a mother whose baby was diagnosed with a spinal defect]** “I normally hear people say that the scan reduces your years, that if you keep on going to the scan, your years keep on reducing but me I don’t believe in that” **[Interview with a mother who had a scan but experienced a stillbirth]** “What they are saying, I think they are lies because when I got out, I have never got any problem” **[R4: FGD 1 with mother attending ANC clinic]**

### Views and experiences of mothers

#### Safety

Mothers had mixed views about the safety of the ultrasound scan technology. While some of them had positive feelings about it, nearly half believed that it could harm them or their unborn baby. Women across the consultations (FGDs and individual interviews) repeatedly raised community rumors that the scan reduces their lifespan. Due to these rumors, some women declined the ultrasound examination. Of the women that undertook the scan, some were afraid before enrollment but felt safer after receiving educational talks about it.

The truth is I know very well that when you put your child in the scan, the child won’t be delivered like a clever child. There is a way that electricity affects the child’s brains and he or she becomes dull. So, I said to myself, instead of delivering of a dull child, I would rather not go for the scan [**R1: FGD with mothers who declined an ultrasound examination**].

I fear the scan, if I don’t have a very serious disease, I can never go for a scan. Because they say that the scan reduces peoples’ years (life expectancy) [**R3: FGD 2 with mothers attending routine ANC**].

I had a lot of thoughts but when they educated me about them (safety of ultrasound), I stopped [**Interview with a mother whose baby was diagnosed with a spinal defect**].

I normally hear people say that the scan reduces your years (life expectancy), that if you keep on going to the scan, your years keep on reducing but me I don’t believe in that **[Interview with a mother who had a scan but experienced a stillbirth]**.

#### Resource availability

##### Ultrasound service availability

Some mothers attending FGDs said they frequently failed to access the ultrasound services at Kagadi Hospital due to power outages or machine breakdown. The power outages during the study period sometimes lasted for nearly two weeks, and twice damaged the project equipment. This halted ultrasound services for several weeks and many women missed their follow-up Doppler examinations. One of the mothers who suffered a neonatal death said she was unable to access a follow-up ultrasound to check the status of her pregnancy.

When I came back, there was no power, so I returned (home) without going to the scan. Since then, I have been coming and finding no power. I don’t know if I will get a chance of getting scanned today [**R5: FGD 1 with mothers undergoing routine ANC**].

Yes, they told me to go back on 10^th^ of July 2019 but when I went there, they told me that the scan is dead and we came back (home) [**interview with mother who suffered a neonatal death**].

#### Partner involvement

Partner involvement emerged as a key issue during the discussions, and we further probed mothers about their spousal involvement in antenatal care and access to ultrasound services. The majority of the women who underwent ultrasound examinations saw their spouses as very supportive. In addition, our team observed many men accompany their spouses to the scan appointments.

Like me, I was with my husband and they told me to go for a scan. He also accepted and I came [**R5: FGD 1 with mothers who delivered in the hospital**].

When I heard about the service, I told him and we started from there to come together [**R0: FGD 3 with mothers who delivered in the hospital**].

I came with my husband and I told him immediately after the scan about the baby. And when we reached home, we explained to my mother-in-law about all the results they gave us [**R3: FGD2 of mothers who delivered at home**].

### Views and experiences of providers

#### Safety

Most of the healthcare workers said mothers believed ultrasound examinations would reduce their lifespan. This misconception was common and thought to be due to a lack of differentiation between ultrasound and X-ray. Before recruitment into the EPID study, all mothers attending the antenatal care clinic received educational talks about obstetric ultrasound to alleviate their fears.

Most people here associate the scan with reducing years (life expectancy) [**Interview: midwife 5**].

Sometimes they tell you, it reduces our age [**Interview: Sonographer 1**].

Most women in the villages dislike this scan. They think that the scan chops their years [**Interview with midwife 1**].

### Resource availability

#### Ultrasound service availability

The majority of the healthcare workers said power blackouts and sudden breakdowns of the ultrasound equipment due to electricity excesses were serious impediments to the continuity of the scan services at the hospital.

We had a challenge, a serious one, and mothers would wait, we would all be waiting for power; when it comes, it comes and it goes [**Interview with midwife 5**].

But the way I see, people really like the scan, they like coming here, even today, mothers came for Doppler and we told them we are sorry that our machine went for service [**Interview with Sonographer 2**].

Generally, such advanced ultrasound equipment is not readily available in remote health facilities in rural Uganda due to the high costs, and this was the first of its kind in Kagadi Hospital. An administrator in Kagadi Hospital said that many healthcare workers were unfamiliar with Doppler ultrasound technology, image analysis and using diagnostic test results to inform clinical decisions.

It is a huge advancement, which, of course, very many people are not aware of, especially the Doppler aspect of it. You know people only know scan, scan, but this advanced scan, many people are not aware of it and of course we were not expecting it, given the finances involved [**Interview with an administrator 1**].

#### Technicalities aspect of Doppler exams

The majority of the healthcare workers expressed concerns over the complexity and duration of the Doppler ultrasound examinations. The sonographers reported that the mother’s habitus, health condition and level of fetal activity influenced the duration of the exam. Longer procedures caused delays in the ultrasound scan unit.

I’m not technical in these things of the scan, but what I have seen when they are doing a Doppler, they take a lot of time…dating is okay but when they are doing Doppler, it takes a lot of time [**Individual interview with midwife 5**].

Sometimes also, it takes time to get the vessels, as you are getting there, a mother says I’m tired; you wait and I change, like that. Though others are fast and they don’t take a lot of time [**Individual interview with Sonographer 1**].

#### Follow-up of pregnant women

Most of the healthcare workers found it challenging to follow-up pregnant women until delivery. Some mothers lacked mobile phones, while others resided too far away and lacked funds and/or means of transport to and from the hospital. Therefore, necessary close monitoring of high-risk pregnancies using follow-up Doppler assessments was impractical for some mothers in the study setting.

Some mothers have no contact [**Interview with midwife 5**].

Another thing, may be, is that these mothers complain of distance if you tell them to come back for Doppler [**Interview with midwife 1**].

The midwife can write that I have referred this mother; I have identified a transverse lie. And reaching Kagadi in ultrasound, giving the results to the mother that your baby is okay and not in a transverse lie, the mother can say if ultrasound is saying my baby is in good position, I can stop coming** [Interview with an administrator 2].**

## Discussion

This study assessed the views and experiences of women, healthcare workers, and health system managers regarding the use of modern ultrasound equipment with Doppler and color flow capabilities in an obstetric department in a rural Ugandan hospital. Many women had never seen or undergone an ultrasound scan and the majority of them were afraid it would harm them or their fetuses. On a positive note, the majority of the women found their husbands supportive of antenatal care attendance including the use ultrasound services. Healthcare providers were unfamiliar with Doppler technology and using it to guide clinical decisions. Other barriers to its implementation were a shortage of trained local staff, insufficient equipment, long distance to and from the hospital, and frequent power cuts.

Mothers felt that an ultrasound exam would reduce their lifespan and/or harm their fetuses. Nearly every healthcare worker we interviewed had heard this. The literature reports mixed perceptions of women regarding ultrasound safety [14]. Women in selected health facilities in Uganda have previously reported fears and misconceptions about imaging [15]. Similarly, in Thailand, 5.1% of respondents reported that they believed ultrasound could be dangerous, while the majority viewed it as a safe and useful tool in pregnancy [11]. In Kenya, 30% (10/34) of the women interviewed before receiving an ultrasound were worried it could harm them or their fetus [10]. That proportion dropped to 8% (n = 2/25) at their second or subsequent ANC visit [10], demonstrating that with exposure and proper health education, perceptions can transform. In a high-income setting, women generally held positive views about getting a third trimester ultrasound [20]. Even though some women in this study were afraid that the procedure could cause harm, the service demand remained high probably due to the larger number of patients Kagadi hospital receives yet they had only a single machine donated by the EPID project.

Negative views may be attributable to lack of exposure and common myths. Such misconceptions regarding the safety of ultrasound in pregnancy could preclude future adoption and large-scale implementation of this technology in vulnerable, poor communities. However, it is also possible that widespread implementation and continued public engagement on the safety and role of obstetric ultrasound, stressing the importance of early initiation of antenatal care and adequate pregnancy dating can lead to its greater acceptance over time.

Pregnant women had challenges accessing screening and follow-up scans due to the unreliable electricity supply characterized by frequent blackouts, equipment breakdown attributed to power supply, insufficient equipment, shortage of trained local staff and long distance to and from Kagadi Hospital. Empowering community-level heath workers to support expectant mothers and engaging them with the health system could improve follow-up care. Uganda’s skilled birth attendance policy previously implemented by the Ministry of Health to improve access to obstetric care through training more health workers, expanding infrastructure, equipment, and distribution of supplies could be enhanced [21]. We recommend context-specific strategies to improve access to follow-up care in other LMICs.

In addition, the majority of the women had never seen or undergone an ultrasound examination. These findings are in line with the results of previous studies undertaken in similar low-resource settings [7, 8, 12–14], implying inequitable distribution of ultrasound services and its benefits to vulnerable poor women in rural communities. This is contrary to the WHO recommendations that every woman should receive at least one scan before 24 weeks gestation to estimate gestational age and improve detection and referral for the care of pregnancy complications [4]. Accurate gestational age estimation and early identification of complications guide the timing for delivery and appropriate management of a mother and fetus at risk [4]. Doppler ultrasound is indicated for close monitoring and management of high-risk pregnancies [4].

However, the healthcare staffs’ interpretation and application often limit the usefulness of a diagnostic tool such as Doppler ultrasound. Healthcare staff in Kagadi Hospital were unfamiliar with antenatal Doppler ultrasound technology and using it to manage high-risk pregnancies, but received training about it and a Doppler ultrasound machine from EPID project. There is a need for training activities beyond Kagadi Hospital. Our study highlights the need for continued education and targeted interventions on the interpretation of Doppler ultrasound findings as an important component of introducing modern ultrasound technology for clinical practice in Uganda and beyond. Likewise, two studies from Uganda and Tanzania observed that frequent training may be necessary when introducing new obstetric tools into ANC settings of LMICs to improve healthcare providers’ knowledge and ensure their acceptability and correct usage, despite differences in the technologies studied [22, 23]. Furthermore, local staff and stakeholders should be involved in developing realistic clinical practice guidelines (for example “bottom-up guidelines” in Suriname) so that the new interventions are well suited and accepted in the local setting (for example the Partoma project in Tanzania) [24, 25]. It is key to note that in many LMICs, babies still die due to poor transportation, lack of skilled health care workers, poor quality of care provided to pregnant and laboring women, and high rates of home deliveries among other reasons. Therefore, while introducing advanced ultrasound machines to weak health systems, we must carefully consider where it is likely to be beneficial. Its introduction into antenatal care requires a health system strong enough to manage an increase in the number of detected high-risk pregnancies including the surgical capacity to manage a potential increase in caesarean sections.

In the current study, the majority of the women reported that their husbands supported them to attend ANC and use ultrasound services. Male involvement in sexual and reproductive health has recently been recognized as a strategy for enhancing ANC attendance and utilization of antenatal care interventions [26, 27]. Engaging male partners and other stakeholders to support women and children to access care promote men’s positive involvement as husbands, fathers, and birth companions [26–28]. Men in Uganda have most of the access to economic resources and decision-making power, and their optimal involvement could facilitate the implementation and uptake of ultrasound. However, key actors such as international organizations and the Ministry of Health inadequately address male involvement strategies in Uganda, and gaps between policy and practice exist [29]. Strategies that accommodate men, such as making the obstetric services more father-friendly by improving ultrasound room spaces, and male recruitment into healthcare services are required.

Strengths of this study included the large number of interviews conducted with a heterogeneous sample including women, healthcare workers and health system managers, yielding broader understandings of Doppler ultrasound implementation issues in a low resource setting from the perspectives of major actors in maternal, newborn, and child health. Additionally, a multi-disciplinary and team approach in developing a working analytical framework, selecting emerging codes and themes, and data interpretation allowed multiple stakeholders to engage with the data and offer their perspectives during analysis. This systematic and rigorous data analysis approach in addition to triangulation of results from different methods and sources enhanced the credibility and trustworthiness of our findings.

The study had some limitations. We had one main coder (OK) though she was very experienced in qualitative research and the codes were continuously reviewed and discussed by a larger research team to ensure that no important perspectives were left out. Although interviews at different time points (first contact, follow-up Doppler exam, and after delivery) and multiple facilities, and sustained engagement with respondents to capture their views on our interpretations might have provided additional insights, this is one of the first studies of its kind. Further, we did not conduct any IDI of healthy women but many women of this category were included in FGDs. While practitioners should extrapolate the findings to other geographical regions with caution, we remain confident that they are representative of similar low resource settings. Important dimensions outside the scope of the current study such as views of men could be considered in future research.

## Conclusions

This study found limited exposure to Doppler ultrasound technology among women and healthcare providers in mid-western Uganda. Partner involvement may potentially promote its acceptance and use to improve the health of women and their unborn babies. Some implementation challenges included difficulty in offering follow-up care to mothers detected with complications and Doppler ultrasound required a high level of training.

It is critical to seek family and community engagement to break the associated myths and misconceptions, and to strengthen the healthcare system to improve access to necessary interventions and follow-up care for complications detected at the ultrasound exam. Lastly, while introducing advanced ultrasound machines to weak health systems, adequate training of healthcare professionals is important to avoid the risk of unnecessary interventions based on misinterpretation of the findings and carefully consider where it is likely to be beneficial. It should be embedded in the local setting with realistic clinical practice guidelines.

## Supplementary Information

**Additional file 1:** FGD guide for women

**Additional file 2:** Interview guide for women

**Additional file 3:** Interview guide for healthcare workers

**Additional file 4:** Interview guide for healthcare managers

## Data Availability

The datasets used and/or analyzed during the current study are available from the corresponding author on reasonable request.

## Abbreviations

ANC: Antenatal care
LMICs: Low- and middle-income countries
HICs: High-income countries
WHO: World Health Organization
COREQ: Consolidated criteria for reporting qualitative research
FGDs: Focus group discussions
R: Respondent
