# Supplementary material for: Antenatal Doppler ultrasound implementation in a rural sub-Saharan African setting: exploring the perspectives of women and healthcare providers
Source: Reprod Health. 2021 Oct 7;18:199. doi: 10.1186/s12978-021-01233-5 (PMC8499453; doi:10.1186/s12978-021-01233-5)
Supplement: Supplementary file 1 — Additional file 1: FGD guide for women [file 12978_2021_1233_MOESM1_ESM.pdf]

FOCUS GROUP DISCUSSION GUIDE FOR WOMEN (ENGLISH VERSION)

[*Pregnant women seeking ANC services, women who deliver from home, and women who maintain all ANC contacts and deliver from the hospital*]

Study Title: Understanding women's and healthcare workers' experiences and perceptions regarding the use of Doppler Ultrasound Examination for Pregnant Women in Western Uganda

- Age of study participants
- Marital status of participants
- Education level of participants
- Number of previous pregnancies, including the current one.
- Age of pregnancy at first scan (months)

Opening question

1) How do people in the community describe pregnant women?

Questions on awareness

2) How would you describe ultrasound scan to a person who doesn't know anything about it?
   - What you were told about the role of ultrasound scan in pregnant women
3) How did you learn about ultrasound?
   - Sources of information about ultrasound scan?
   - Had you heard about scan before it was offered at Kagadi hospital?
   - Did you previously know that Ultrasound Scan was available at the Kagadi hospital before it was offered?

Perceptions and feelings about USS

4) How did you feel when you learnt that there was ultrasound scan while you were already at the hospital?
   - Were you happy, sad, scared, not prepared, not sure, etc.?
   - Explain your imaginations and expectations before you underwent the scan
   - Were you given the opportunity to decide whether to use ultrasound scan or not?
   - If yes, how much time were you given to decide to do a scan?
   - Probe if they needed to consult their male counterparts, elders, etc.
   - Find out if ultrasound scan was compulsory for pregnant women
5) Describe the process of undergoing an ultrasound scan examination
   - Who did the scan (Would you have preferred a man/woman to do ultrasound scan)?
   - Did you feel comfortable if it was a man? And why?
   - What exactly happened during the scan
   - How did it feel to undergo a scan? Did you enjoy it, get scared, felt pain etc.?
6) Describe the aspects of ultrasound scan that you enjoyed during your examination
   - What aspects did you enjoy and why?

- Which aspects didn't you like and why?
- Explain how the aspects that you didn't enjoy can be improved?

7) How would you describe what happened in relation to what you had imagined or expected?
8) Describe how this process changed the way you perceived your antenatal visits and ultrasound scan in general?

Communication/Relationships with HCWs

9) Explain what the HCW discussed before and after the scan
   - Safety of the ultrasound scan
   - Health of the baby
   - Expected date of delivery, do you think/ was it accurate at the time of delivery
10) How did it feel to learnt about your baby's delivery date? feelings about the baby…happy sad, anxious, did they feel the baby wasn't safe etc.).
    - Who did you discuss your feelings with (HCW, family member, traditional healer)
    - How did they react to your fears? Were they helpful and why?
11) How would you describe the staff and place where the scan was conducted (friendliness, warm HCWs, ultrasound machine etc.)?

Barriers and enablers to completing ANC Visits

12) How do people in the community describe an ultrasound scan for pregnant women?
    - Why do they think about USS like that? Where do they get this information?
    - Do you always agree with what they think? If yes/no, why?
13) In other communities, some mothers perceive ultrasound scan to be harmful to their health and the health of their babies and therefore feared to complete their ANC visits.
    - Explain your thoughts in relation to this
    - Give other reasons for non-completion of ANC visits/ not delivering at the hospital?
    - Probe for reasons for the answers given
    - Why do other women come complete their ANC visits and deliver at the hospital after ultrasound scan?
14) If you came across a pregnant woman, would you recommend her to undergo an ultrasound scan?
    - Why would you volunteer to do that?
    - Can you give me a scenario of what you would tell this person?

Thank you for your time!

FOCUS GROUP DISCUSSION GUIDE FOR WOMEN (RUNYORO VERSION)

Ebikaguzo ebirakozesibwa mukuhanuura okwahamu (Abakazi abaine enda abaizire kutunga obuhereza bw'okukeberwa, abakazi abazaaliire muka, abakazi abasoboire kwija emrundi y'okukeberwa yoona kandi bazaarra omwirwarro)

Study Title: Okwetegereza Eby'abakazi Hamu N'abajanjabi Arabamu Kandi N'ebitekerezo Ebirukukwata Ha Kukozesa Akativi Okukebere Abakazi Abaine Enda Omubugwa Izooba Bwa Uganda

- Emyaka y'omweteranizi
- Embeera y'omweteranizi muby'obuswezi
- Eby'okusoma by'omweteranizi
- Omuhendo gw'abaana otaireho nenda eyahati
- Emyezi y'enda nambere wakeberiirwe akativi omurundi gw'okubanza

Ekikaguzo eky'okukinguraho

15) Abantu bomubyaaro abakazi benda babeeta rundu babazaaho bata?

Ebikaguzo hakumanya

16) Okusobora ota okusoborra akativi hali omuntu ataine ekyakamanyireho?
    - Inywe mukagambirwaaki hamugaso gwaka tivi hali abakazi benda?
17) Mukaija muta okumanya hakativi
    - Amakuru agakwata hakativi nambere garuga
    - Mukaba muhuliire ha kativi obu baali batakandikire kukakozesa hairwarro lya Kagadi?
    - Mukaba mukimanyire ngu akativi karoho hairwarro lyakagadi obubali batakakibagambiire hanu?

Ebitekerezo hamu n'okwehurra hakativi

18) Mukehurra muta obu mwamanyire ngu hairwarro hanu nibakozesa akativi kandi obu mumazire kuhikaho?
    - Mukasemererwa, mukabihirwa, mukatiina, mukahurra timwetegekere, mukaba mutakugumya kurungi?
    - Munsoborre ebimwaali nimutebereza kandi nimunihira mutakagenzire mukativi
    - Mukaheebwa omugisa okucwamu okukuzesa akativi rundi nangwa?
    - Obu kiraba ngu ego, mukaheebwa bwiire oburukwingana nkaha okusobora okucwaamu?
    - Kaguliriza obu baraba bayetagisize okwehanuzaaho nabasaija baabo, abakuru, nabandi
    - Zoora akativi obu karaaba kaali kabuli muntu wena anyakwiine enda.

19) Munsoboroorre emitwaarre ey'okugenda n'okukorwaaho akativi okekeberwa enda
    - Nooha ayabakozireho mukativi (mwakugondeze omukazi rundi omusaija okukebeza akativi?
    - Obu muraaba mwakozirweho omusaija, mukehuurra kurungi? Kandi habwaaki?
    - Kiki kyenyini ekyabaireho obumwaali mukativi akakebere enda?

- Mukehurra muta kukeberwa nakativi? Mukakegondezaamu? Mukatiina, mukahuurra obusaasi

20) Munsoborre ebicweka ebyakativi ebimwakizire kwegondezaamu obubaali nibababakebera
    - Bicweka ki ebyabasemiize kandi habwaaki?
    - Bicweka ki ebitarakusemiize kandi habwaaki?
    - Munsobororre nkuku tusobora kusemeza ebiceka ebyo ebitarabasemiize

21) Musobora kunsoboroorra muta ebyabaireho okusigikiirra hali ebimwaali nimunihira?
22) Munsobororre nkoku okukeberwa kunu kwahingisizeemu omulingo waroziremu kandi watekerezaamu okwija mwirwarro kukeberwa kandi nokukozesa akativi okutwarra hamu?

Okubaza hamu n'enkwatagana nabajanjabi

23) Munsobororre biki abajanjabi ebibabagambiire mutakagenzire mukativi kandi nobu mwali murugireyo?
    - Safety of the ultra sound scan
    - Akativi okuba katakuleeta kizibu kyoona
    - Obwomeezi bwomwaana ali munda
    - Ebiro ebikuteberezibwa kuzaraamu, bikaba nibyo hakiro wazaliremu?
24) Mukahurra muta okumanya Ebiro abaana banyu baali nibagenda kuzalirwamu? Mukahaurra muta hali omwana, kusemererwa, kubihirwa, kweralikirra, mukehurra muta ngu omwana tali kurungi?
    - Nibaha abamwagaibe nabo nkoku mwali nimwehurra (abajannjabi, omuntu womuka, omufumu)
    - Bakahurra/ bakagarukamu bata hali okutiina kwawe? Bakaba bomugaso? Kandi habwaki?
25) Ekikaro nambere okukeberwa akativi kwakoliirwe hamu nabajanjabi ababakebiire musobora kubansooborra muta? (bagonza abantu, abajanjabi nibakwata kurungi, akativi nebindi)?

Ebitukutangira nokuyamba kumaliriza emirund eyokukeberwa

26) Abantu b'omubyaro akativi k'okukebera abakazi benda bakasoborra bata?
    - Habwaki batekereza bati ha kativi? Amakuru ago bagaiha nkaha?
    - Do you always agree with what they think? If yes/no, why?
    - Nainywe mwikirizanganiza nabo hali ebibatekereza? Obukiraaba ego/nangwa, habwaki?
27) Mubyaro ebindi, abakazi batekereza akativi kasobora kubakobuzibu hali mama n'omwana alimunda nahabweeki batiina kwija kukeberwa emirundi yoona.
    - Munsoborre ebitekerezo byanyu hali ekintu kinu
    - Mumpeyo ensonga ezindi ezireta okutamaliriza okukeberwa enda nokutazaarra omwirwarro
    - Weyongere okukaguza habigarukwamu ebibahaireyo

- Habwaki kandi abakazi abandi bamarayo okukeberwa kandi bazaarra omwirwarro hanyuma yokukeberwa nakativi

28) Kakusangwa otangaana omukazi aine enda, okusobora kumuha amagezi agende mu kativi
    - Habwaki mukusobora kukikora?
    - Mukusobora nkumpayo ekyokurorraaho eky'ebyo enimusobora kumugamba?

Webale obwiire bwaawe!
