# Supplementary material for: Antenatal Doppler ultrasound implementation in a rural sub-Saharan African setting: exploring the perspectives of women and healthcare providers
Source: Reprod Health. 2021 Oct 7;18:199. doi: 10.1186/s12978-021-01233-5 (PMC8499453; doi:10.1186/s12978-021-01233-5)
Supplement: Supplementary file 2 — Additional file 2: Interview guide for women [file 12978_2021_1233_MOESM2_ESM.pdf]

INTERVIEW GUIDE FOR WOMEN (ENGLISH VERSION)

Study Title: Understanding Women's and Healthcare Worker's Experiences and Perceptions Regarding the Use of Doppler Ultrasound Examination for Pregnant Women in Western Uganda

SECTION A: Interview Guide for Women (Refused to undergo ultrasound Examination)

- Age of study participants
- Marital status of participants
- Education level of participants
- Number of previous pregnancies, including the current one.
- Age of pregnancy at first scan (months)

1) Tell me what it is like being pregnant?
   - How does it feel?
   - How do people in the community describe or think about a pregnant woman?
2) Describe the services that you were offered during your (antenatal care) ANC visit
   - Health education, HIV testing and counselling, ultrasound scan services, call or home visits (later after the visit)
   - How did staff treat you during your ANC? (friendliness, warm HCWs, kind, sympathetic)
3) Tell me more about ultrasound scan. How did you learn about it?
   - Had you heard about an ultrasound scan before it was offered at the hospital? If yes, from where?
   - Did you previously know that ultrasound scan was available at the hospital before it was offered to you?
   - What did HCWs say the role of ultrasound scan was?
   - Were you given the opportunity to decide or it was compulsory?

4) How did you feel when you learnt that ultrasound scan was available and free at the hospital?
5) How did you feel after the HCW explained the process of undergoing an ultrasound scan?
6) What did you decide? Why did you decline to undergo the ultrasound scan even after the HCW explained that it was safe? Did you have enough time to ask questions? What discussion did you have with the healthcare workers after you had declined? How much time did you need to decide?
7) Explain what happened after you had declined the scan (feelings, who did you discuss your experience with? Did you tell other people about it) did you continue to come for ANC? In your opinion what could have been the benefits if you had undergone the ultrasound scan?
8) If there was another chance of undergoing the scan, what would be your reaction?

SECTION B: Interview Guide for Women (Pregnancy complications/Lost their babies 4 weeks after delivery)

- Age of study participants
- Marital status of participants
- Education level of participants
- Number of previous pregnancies, including the current one.
- Age of pregnancy at first scan (months)

1) Tell me what it is like being pregnant?
    - How does it feel?
    - How do people in the community describe or think about a pregnant woman?
2) Describe the services that you were offered during your ANC visit
    - Health education, HIV testing and counselling, scan services, call or home visits (later after the visit)
    - How did staff treat you during your ANC? (friendliness, warm HCWs, kind, sympathetic)
3) Tell me more about ultrasound scan. How did you learn about it?
    - Had you heard about scan before it was offered at the hospital? If yes, from where?
    - Did you previously know that ultrasound scan was available at the hospital before it was offered to you?
    - What did HCWs say the role of ultrasound scan was?
    - Were you given the opportunity to decide or it was compulsory?
4) How did you feel when you learnt that ultrasound scan was available and free at the hospital?
    - Did you decide to undergo a scan or it was mandatory for all pregnant woman?
    - If not mandatory how much time did it take you to make a decision to undertake a scan?
    - What were your expectations before you underwent the scan?
5) Tell me about the actual day you underwent the ultrasound scan examination
    - Who did the scan (Would you have preferred a man/woman to do ultra sound scan?)
    - Did you feel comfortable if it was a man doing scan? And why?
    - How did you feel when you underwent the actual ultrasound scan? Did you enjoy it, get scared, felt pain?
6) Describe the aspects of ultrasound scan that you enjoyed during your examination
    - What aspects did you enjoy and why? Which aspects didn’t you like and why?
    - Explain how the aspects that you didn’t enjoy can be improved?
    - As you underwent the scan, did it meet your earlier expectations in relation to the role of the scan as earlier told by the HCW?
    - How did you feel after the scan?

7) Describe the outcome of the scan that was communicated to you
   - The baby had complications
   - The baby was dead
   - The baby was okay
8) For those who lost babies within 4 weeks after birth: How did you feel about the results from the scan?
   - How did it feel to learnt about your baby's delivery date? feelings about the baby…happy sad, anxious, did they feel the baby wasn't safe etc.)
   - What do you think was not done well that could have caused the baby to die after?
9) For those who had complications: How did you feel after getting the news about the complication?
   - Who did you share the outcomes with?
   - How did they react to your fears? Were they helpful and why?
   - What ideas do you have about what could have caused the complications?
   - In your opinion, explain what could have caused the pregnancy complications that you had/have?
10) If still pregnant, what is currently being done about your condition?
   - How confident are you about the current interventions to ensure that you and the baby continue to live?
   - Is there anything that you think the project could do to improve this pregnancy outcome? If the baby died, what would the hospital have done to ensure that your baby survived?
   - How did your experience of the ultrasound scan shape the way you perceived your antenatal visits now or in the future?
11) How do people in the community describe a scan for pregnant women?
   - Why do they think about ultrasound scan like that? Where do they get this information?
   - Do you always agree with what they think? If yes/no, why?
12) Some of the women I interviewed earlier told me that they stopped going for ultrasound scan because they felt the scan would be harmful to their health and the health of their babies
   - What do you think about this idea? Why?
   - What are some of the reasons why women would not complete their ANC visits or deliver at the hospital?
13) If you came across a pregnant woman, would you recommend her to undergo an ultrasound scan?
   - Why would you volunteer to do that given the outcome of your pregnancy?
   - What aspects of ultrasound scan would you emphasize?
14) Apart from the ultrasound scan, explain what you think the hospital can do to ensure that pregnancy complications are detected early and ensure babies survive.

Thank you for your time!

INTERVIEW GUIDE FOR WOMEN (RUNYORO VERSION)

Study Title: <u>Okwetegereza Eby'abakazi Hamu N'abajanjabi Arabamu Kandi N'ebitekerezo Ebirukukwata Ha Kukozesa Akativi Okukebere Abakazi Abaine Enda Omubugwa Izooba Bwa Uganda</u>

EKICWEKA A: Ebikaguzo ebirakozesibwa hakukaguza abakazi (Abakazi abayangire kukozesa akativi)

- Emyaka y'omweteranizi
- Embeera y'omweteranizi muby'obuswezi
- Eby'okusoma by'omweteranizi
- Omuhendo gw'abaana otaireho nenda eyahati
- Emyezi y'enda nambere wakeberiirwe akativi omurundi gw'okubanza

9) Ngambira, nikisisana ki okuba nenda?
    - Kihulirwa kita?
    - Abantu b'omukyaro batekerezaaki rundi bagambaki habakazi benda?
10) Nsobororra obuhereza obu wahairwe obuwaizire hairwarro kukeberwa
    - Okusomesebwa, okukeberwa akahuka ka silimu, kukeberwa akativi, obuhereza bwesimu, kubungirwa nebindi)
    - Abajanjabi bakakurabya bata obuwaizire kukeberwa? (bagonza abantu, nibatangiirra, baine ekisa)
11) Weyongere okungambira ha kativi. Okakamanyira nkaha?
    - Okaba okahuliireho obubaali batakakugambiire hanu hairwarro? Obu kiraba ego, nkaha?
    - Okaba okimanyire ngu kativi kali hairwarro obubaali batakakugambiire?
    - Abajanjabi bakababagambira akativi kaine mugaso ki?
    - Okaheebwa omugisa kwecweramu rundi buli mukazi aine enda akabaine okukagendamu?
12) Okehurra ota obuwahuliire ngu haliyo akativi kandi kabusa?
13) Okehurra ota obu abajanjabi bamazire kukusoboroorra emitwalize ey'okugenda mukativi?
14) Iwe okasaraho ki? Habwaki wayangire okukeberwa akativi nahanyum y'omujanjabi okukusoboroorra ngu tikaine kizibu? Okaheebwa obwiire okukaguza ebikaguzo? Kuhanuuraki okuwabairenakwo hanyuma y'okwanga? Okaba noyenda obwiire obukwingana nkaha okusobora okucwamu?
15) Nsobororra ekyabaireho hanyuma y'okwanga okugenda mukativi (okwehuurra, nooha owu wagabaineho nebyo ebiwarabiremu? Oine owu wakigambiireho? Okeyongera kwija kukeberwa okuruga obwiire obu? Mukutekereza kwawe, birungi ki ebiwakutungire kakuba wali iokiriize kukeberwa akativi?
16) Kakuba otunga omugisa ogundi okukeberwa akativi, nosobora kugarukamu ota?

EKICWEKA B: Ebikaguzo ebirakozesibwa hakukaguza abakazi (Enda zikaba n'ebizibu/ bakafeerwa abaana sabiiti ina hanyuma yokuzaara

- Emyaka y'omweteranizi
- Embeera y'omweteranizi muby'obuswezi
- Eby'okusoma by'omweteranizi
- Omuhendo gw'abaana otaireho nenda eyahati
- Emyezi y'enda namebere wakeberiirwe akativi omurundi gw'okubanza

1) Ngambira, nikisisana ki okuba nenda?
    - Kihiirwa kita?
    - Abantu bomukyaro abatekerezaaki rundi bagambaki habakazi benda?
2) Nsobororra obuhereza obu wahairwe obuwaizire hairwarro kukeberwa
    - Okusomesebwa, okukeberwa akahuka ka silimu, kukeberwa akativi, obuhereza bwesimu, kubungirwa nebindi)
    - Abajanjabi bakakurabya bata obuwaizire kukeberwa? (bagonza abantu, nibatangiirra, baine ekisa)
3) Weyongere okungambira ha kativi. Okakamanyira nkaha?
    - Okaba okahuliireho obubaali batakakugambiire hanu hairwarro? Obu kiraba ego, nkaha?
    - Okaba okimanyire ngu kativi kali hairwarro obubaali batakakugambiire?
    - Abajanjabi bakabagimbira akativi kaine mugaso ki?
    - Okaheebwa omugisa kwecweramu rundi buli mukazi aine enda akaba aine okukagendamu?
4) Okehurra ota obuwamanyire ngu hairwarro haroho akativi?
    - Obukaraaba kakaba kataine kugendwamu bulimukazi weena, okatwara bwireki okucwamu okugenda mukativi?
    - Biki eby'okaba nonihira otakagenzire mukativi?
5) Ngambira hakiro kyenyini obuwagenzire mukativi
    - Nooha ayakukozireho omukativi? (wakugondeze omusaija rundi omukazi okukukebera nakativi)
    - Wakwehuliire kurungi kakuba ayakukozireho yali musaija, kandi habwaki?
    - Okehurra ota obugenzire mukativi? Kakakusemeza, kakutinisa, kakusaasa?
6) Nsobororra ebicweka byakativi ebyakizire kukusemeza obu baali nibekukebera
    - Bicwekaki ebiwagondeze kandi habwaki, bicwekaki eby'otaragondeze kandi habwaki?
    - Nsobororra nkoku ebicweka eby'otaragondeze bisobora kusemezibwamu
    - Obuwali omukativi, kakuyamba okuhikiriza ebintu ebiwali nonihira otakakagenziremu ebikwataine nomugaso gwakativi omujanjabi ebiyali akugambireho?
    - Okehurra ota hanyuma yakativi

7) Ninkusaba ongambire biki ebyakugambiirwe ebyarugire omukukeberwa akativi
    - Omwana akaba aine obuzibu
    - Omwana akaba afire
    - Omwana akaba ali kurungi
8) Baferiirwe abaana hanyuma yasabiiti ina: Okehurra ota hanyuma y'okutunga amakuru agarugire omukativi?
    - Kikakuhuliza kita okumanya ebiro by'okuzalirwamu kwomwana wawe? Kwehurra hali omwana,…nkasemererwa, kweralikirra, okehurra ota omwana obu araba yali atali kurungi)
    - Notekereza kiki ekitarakozirwe kurungi ekisobora kuba kyarugiireho omwana kufa hanyuma?
9) Abaine enda baitu abaana baine ekizibu: Okehurra ota hanyuma y'okutunga amakuru ngu omwana aine ekizibu?
    - Amakuru ago okagabaganaho nooha?
    - Bakagarukamu bata hakutiina kwawe, bakasobora kukuyamba kandi bata? Oine bitekerezo ki ebikukwata ha kiki ekisobora kuba kyaleteriize obuzibu buno omwana obu aine/yali aine?
10) Obu araba akyaine enda, kiki ekiriyo nikukorwa handa yaawe? Nogumya ota ngu ebiriyo nibikorwa hati okurora ngu omwana yayongere kwomeera?
    - Haroho ekintu kyoona okyokutekereza ngu entegeka enu nesobora kukora okusemeza ebiraruga munda yaawe? Omwana obu araba yafiire, kiki irwarro ekiryakukozire okurora ngu omwana wawe akomeera?
    - Hanyuma yokukozesa akativi, kikahindura kita entekereza yawe hakugenda kukeberwe hato rundi omumaiso?
11) Abantu b'omukyaro basoborra bata hakativi akakebera abakazi abaine enda?
    - Akativi bakatekerezaho ki? Amakuru ganu bagaiha nkaha?
    - Oikiranganiza nabo hali ebibakutekereza? Obukiraba ego/nangwa,habwaki?
12) Abamu habakazi abanakagwiizeeho kara bakangambira ngu bkaleka kugenda mukativi habwokuba bakabe nibatekereza nikasobora kuletera mama n'omwana obuzibu.
    - Iwe kinu nokitekerezaaho ki? Kandi habwaki?
    - Nsonga ki ezimu ezisobora kuletera abakazi okutamarayo emirundi eyokukeberwa rundi kuzaarra hairwarro?
13) Kakusangwa otangaana omukazi aine enda, okusobora kumuha amagezi okugenda mukativi?
    - Habwaki okusobora kukikora okusigikirra biki ebyakurugiiremu mukutwara enda?
    - Bicweka ki ebyakativi ebosobora kutekaho amaani kumusoboroorra?
14) Oihireho akativi, nsobororra kiki ekyorukutekereza irwarro ekirisobora kukora okurora ngu enda eziroho obuzibu zakeberwa kandi zamanywa kara okurora ngu abaana bayomeera?

Webale obwiire bwawe!
