# Supplementary material for: Antenatal Doppler ultrasound implementation in a rural sub-Saharan African setting: exploring the perspectives of women and healthcare providers
Source: Reprod Health. 2021 Oct 7;18:199. doi: 10.1186/s12978-021-01233-5 (PMC8499453; doi:10.1186/s12978-021-01233-5)
Supplement: Supplementary file 3 — Additional file 3: Interview guide for healthcare workers [file 12978_2021_1233_MOESM3_ESM.pdf]

**INTERVIEW GUIDE_HEALTHCARE WORKERS (ENGLISH VERSION)**

**Study Title: <u>Understanding Women's and Healthcare Workers' Experiences and Perceptions Regarding the Use of Doppler Ultrasound Examination for Pregnant Women in Western Uganda</u>**

- Age of Key Informant
- Job title/position held
- Education level
- Years in current position

You are being invited to participate in the above study. We selected you to participate because of your role and expertise in the area of child and maternal health. The information you will give us will contribute to improved maternal and child health services in Kagadi hospital. We will be asking you questions related to the use of ultrasound examination for pregnant women in Kagadi and the district in general, the barriers to accessing ANC services and ways to improve maternal and child health in the district. Feel free not to answer any questions that you are not comfortable with.

1) What does your role in the EPID project involve?
2) How do women come to learn about ultrasound scan services that you offer in this hospital?
   - Do women come when they already know that ultrasound scan is offered at the facility?
   - How do they learn about such? Probe for sources of information about the scan
3) Describe the process of providing ultrasound examination to pregnant women in this hospital
   - Probe for health education aspects
   - The discussions and activities that take place in the scan room, discussions about the scan
   - How do they react or respond? What is your experience with those that refuse to use the scan?
   - How do you address these concerns?
4) Explain the different outcomes of undergoing a scan. How do you communicate results especially when the mother has complications or the baby is dead?
   - How do these mothers respond? Does this affect the subsequent visits to use the scan and complete the ANC visits?
   - How does the outcome of the scan meet the expectations of the mothers?
5) What is it like providing ultrasound scan services to pregnant women (feelings, discomforts)?
6) In your opinions what are the potential appeals and benefits of ultrasound scan for pregnant women?
   - What aspects are less appealing about the scan?
   - Explain the concerns that you may have about ultrasound scan and why?

7) In your opinion what are some of the potential barriers and challenges to:
   - Acceptance of Doppler ultrasound scan,
   - Completion of ANC visits,

- Delivery at the hospital
- Probe for reasons for their views

8) What are your thoughts about the idea of using Doppler ultrasound scan for pregnant women to identify mothers who may be vulnerable to stillbirth?
9) From your perspective what role could ultrasound play in addressing some of these pregnancy challenges? Probe for how and why
10) What would you recommend as the next steps for improving the use of ultrasound and the EPID project for pregnant women?

**Thank you for your time!**
