# Supplementary material for: Antenatal Doppler ultrasound implementation in a rural sub-Saharan African setting: exploring the perspectives of women and healthcare providers
Source: Reprod Health. 2021 Oct 7;18:199. doi: 10.1186/s12978-021-01233-5 (PMC8499453; doi:10.1186/s12978-021-01233-5)
Supplement: Supplementary file 4 — Additional file 4: Interview guide for healthcare managers [file 12978_2021_1233_MOESM4_ESM.pdf]

INTERVIEW GUIDE FOR HEALTHCARE MANAGERS

(ENGLISH VERSION)

Study Title: <u>Understanding Women's and Healthcare Workers' Experiences and Perceptions Regarding the Use of Doppler Ultrasound Examination for Pregnant Women in Western Uganda</u>

Key Informants Interview Guide

- Age of Key Informant
- Job title/position held
- Education level
- Years in current position/district
- Institution represented
- Job responsibilities relating to Maternal and Child Health

1) What does your role in maternal and child health involve?
2) Describe the projects related to maternal and child health being run in your district
3) What are your thoughts about the idea of using Doppler ultrasound scan for pregnant women to identify mothers who may be vulnerable to stillbirth and streamline a path for early intervention in the shortest time possible?
    - Probe for reasons for their views
    - What additional information could you have regarding the use of ultrasounds scan and follow-up of pregnant mothers?
4) In your opinions what are the potential appeals and benefits of ultrasound scan for pregnant women?
    - What aspects are less appealing about the scan?
    - Explain the concerns that you may have about ultrasound scan and why?
    - One of the concerns is that ultrasound scan affects the health of the mother and baby? Any thoughts?
5) In your opinion what are some of the potential barriers and challenges to:
    - Acceptance of Doppler ultrasound scan
    - Completion of ANC visits
    - Delivery at the hospital
    - Probe for reasons for their views
6) From your perspective, what role could ultrasound play in addressing some of these health challenges? (Probe for how and why)
7) What would you recommend as the next steps for improving the EPID project for pregnant women?
8) Is there anything that you would like to tell me that I have not asked?

Thank you for your time!
